# Crush injuries to the lower limbs at a major UK trauma centre: a retrospective observational study

**DOI:** 10.1007/s00590-024-04164-6

**Published:** 2024-12-21

**Authors:** Essam Rama, Saania Jayawant, James Zhang, Matija Krkovic

**Affiliations:** 1 University of Cambridge, Cambridge, UK; 2 Basildon Hospital, South Benfleet, UK; 3 Addenbrooke’s Hospital, Cambridge, UK

**Keywords:** Crush injury, Crush syndrome, Rhabdomyolysis, Compartment syndrome, Amputation, Trauma

## Abstract

**Purpose:**

Crush injuries result from the physical compression of muscles and may lead to crush syndrome. Early fluid resuscitation and surgical intervention is key. Few studies have reported the outcomes of crush injuries in the non-disaster setting. This retrospective study aims to characterise such cases.

**Methods:**

Patients with lower limb crush injuries were identified from an internal database. Non-crush injuries and patients under the age of 18 were excluded. Types of injuries, management, and complications were extracted.

**Results:**

27 patients were included. The right leg (n = 10) was the most frequently injured site. Mechanisms included being run over by vehicles (n = 10) and being crushed by, between, or inside vehicles (n = 8). Fractures were the most common acute injuries (n = 16), while other injuries included rhabdomyolysis, compartment syndrome and degloving. Fluid resuscitation was required in 17 patients. 58 surgeries were performed on 18 patients, with wound debridement and amputations being common. Complications such as acute kidney injury, hyperkalaemia, and sepsis were noted during hospitalisation. Individuals with injuries to the leg or thigh experienced a greater burden of injury and incidence of in-hospital complications compared to those with isolated injuries to the foot.

**Conclusion:**

Crush injuries in the non-disaster setting show distinct mechanisms and injury patterns. Those with crush injuries to the leg or thigh more closely resemble a patient cohort seen in the disaster setting compared to those with isolated foot injuries.

## Introduction

Crush injuries are those caused by a direct physical crushing of muscles by a compressive force [1]. Crush syndrome describes the systemic manifestations of muscle damage due to crush injuries. Bywaters and Beall first described the clinical course of crush syndrome in patients who had been trapped under buildings during the Blitz [2]. These patients suffered hypovolaemic shock and rapidly deteriorated with myoglobinuric renal failure. The acute kidney injury (AKI) is a result of both the hypovolaemia and the nephrotoxicity of myoglobin, urate and phosphate released from cells [3]. Although crush syndrome predominantly affects the kidneys, the clinical picture may be complicated by acute respiratory distress syndrome (ARDS), disseminated intravascular coagulation (DIC), sepsis, hypothermia, metabolic acidosis, hypocalcaemia and hyperkalaemia which can be arrhythmogenic [1, 4]. Crush injuries have since been reported following natural disasters such as earthquakes and landslides, mining accidents, acts of terrorism, and acts of war [5–7]. Atraumatic causes of crush syndrome have also been described; such as following periods of crush by patients’ own body weight, after stroke or intoxication [3].

The development of AKI in crush patients is a negative prognostic indicator for survival [8]. Early administration of intravenous fluid is critical in preventing kidney failure [9] and successful treatment of AKI can help to prevent mortality [10]. Alkalinisation of urine with sodium bicarbonate has been suggested to protect against the development of renal failure [3, 9].

Crush injuries to the head and torso are often immediately fatal, but crushing of the limbs is often survivable despite devastating musculoskeletal trauma [5]. The legs are more commonly injured than the arms and injuries include: fractures, muscle necrosis, and loss of neurovascular function. Surgical management is often required and includes fracture fixation and leg amputation [7]. The development of compartment syndrome which may require fasciotomy to relieve intracompartmental pressure [11].

Crush injuries are rarer in the civilian setting and are usually caused by road traffic collisions and industrial accidents [5]. Few studies have reported the outcomes of crush injury patients in the non-disaster setting. We present a retrospective observational study of patients presenting to a major trauma centre (MTC) in the United Kingdom (UK) with crush injuries to the lower limbs. The East of England Trauma Network coordinates trauma care across six counties and includes twelve trauma units which are linked to the MTC to provide timely, appropriate treatment [12]. Up to 800 major trauma cases are treated annually. We aim to characterise the mechanism and site of injuries, the nature of musculoskeletal trauma, the initial medical and surgical management, and complications during hospital admission.

## Methods

Patients who sustained crush injuries to the lower limb between March 1st 2015 and April 30th 2023 were identified using ICD-10 codes from a prospectively maintained internal (EPIC EMR) database. This was approved by our ethics review board. Individual patient records were examined and pre-determined parameters were extracted using a standardised spreadsheet. Exclusion criteria included: patients under the age of 18 at time of injury, injuries where the primary mechanism was not a crush, those without follow up, or those where information on management was not available as a result of transfer to another hospital for definitive (e.g. operative) management.

Demographic characteristics included: gender, age and body mass index (BMI). Common comorbidities existing prior to injury were recorded. Mechanism and site of injury were recorded for each patient. Fractures to the lower limb were sorted anatomically and whether they were open (compound) or closed (simple). Additional fractures of the spine, ribs, clavicle, and pelvis were recorded. Associated injuries to the skin and/or muscle included: degloving injuries, rhabdomyolysis, and compartment syndrome. Rhabdomyolysis was defined by an increase in serum creatine kinase (CK) 5 times the upper limit of normal (CK > 1000 IU/L) [13] or by clinical evidence of muscle necrosis intraoperatively. Compartment syndrome was diagnosed clinically with serial limb observations (pain out of proportion to signs on physical examination, pain on passive stretching of limb), intracompartmental pressure monitoring was not routinely used. Limb ischemia (suffered, rather than experienced) was determined by the presence of pulselessness, pallor, delayed capillary refill, limb numbness or coldness at initial neurovascular assessment, or intraoperatively. Ankle-brachial pressure index (ABPI) was not routinely used. Lower limb CT angiograms were used to assess for vascular injury, although detailed results of imaging studies are beyond the scope of this article. Acute kidney injury (KDIGO criteria), hyperkalaemia, metabolic acidosis, sepsis and acute respiratory distress syndrome occurring during the course of hospital admission were recorded. Intravenous resuscitation and surgical management were recorded for each patient.

## Results

35 patients were identified, of which 8 were excluded (6 children, 2 injuries not caused by crush), leaving 27 patients included in our study. The median patient age was 43 (IQR 26–56) and 18 of the patients were male. The most prevalent comorbidities were type II diabetes mellitus and hypertension. Demographic parameters are summarised in Table 1.

**Table 1:** Patient demographics and mechanism and site of injury

Demographic data (n = 27)		Number of patients, n (unless specified)
Male		18
Female		9
Age, years	Median	43
	IQR	30 (26–56)
BMI, kg/m^2^	Median	26.4
	Range	21.5
Comorbidities	Type II diabetes mellitus	4
	Hypertension	4
	Congestive heart failure	2
	Previous myocardial infarction	2
	Atrial fibrillation	2
	Asthma	2
	Chronic obstructive pulmonary disorder	1
	Obesity	1
Site of injury*		
Right thigh		2
Left thigh		1
Bilateral thigh		0
Right leg		10
Left leg		6
Bilateral leg		3
Right foot		7
Left foot		8
Bilateral foot		0
*Mechanism of injury*		
Run over by vehicle	Car	3
	Bus	1
	Lorry	3
	Industrial machinery	3
Crushed by/between vehicles		8
Falling objects		7
Crushed by animals		2

*Regions are defined anatomically e.g. the thigh is the area from the hip to the knee and the leg is the area from the knee to the ankle

**Injuries to right and left leg are recorded both as right leg, left leg and bilateral leg

### Site and mechanism of injury

In this study, the thigh and leg were defined anatomically. The most frequently injured site was the right leg (n = 10), followed by the left foot (n = 8) and right foot (n = 7). Three patients suffered bilateral leg injuries, but there were no patients with crush injuries to both feet. The commonest mechanism of injury was being run over by vehicles (n = 10), followed by being crushed by, between or inside vehicles (n = 8). Site and mechanism of injury is shown in Table 1.

### Types of injuries

Acute injuries due to crushing are shown in Table 2. 16 patients suffered fractures. Fractures of the forefoot occurred most frequently (6 open, 2 closed), followed by the fibula (5 open, 2 closed) and tibia (6 closed). Fractures of the midfoot (3 open) and femur (1 open, 1 closed) were less common. Two patients were found to have fractures of the vertebrae and three patients suffered rib fractures.

**Table 2:** Acute musculoskeletal injuries according to site of injury

Site	Type of injury (number of patients, n = 27)							
	Fracture		Degloving injury*	Rhabdomyolysis	Compartment syndrome**	Limb ischaemia (equivalent score on MESS)		
						+ 1 (reduced pulse, but normal perfusion)	+ 2 (pulseless, paraesthesia, slow capillary refill)	+ 3 (cool, paralysis, numb/insensate)
Any	16		5	7	4	0	2	6
Lower limbs	Open	Closed						
Thigh/Femur	1	1	0	2	1	0	0	1
Tibia	0	6	3	7	3	0	2	2
Fibula	2	5						
Forefoot	6	2	3	0	1	0	0	3
Midfoot	3	0						
*Other*								
Spine	0	2	N/A	N/A	N/A	N/A		
Clavicle	0	1						
Ribs	0	3						
Pelvis	0	2						
Other	0	2						

MESS = Mangled Extremity Severity Score (Limb ischaemia scoring: 1 = reduced pulse, but normal perfusion. 2 = pulseless, paresthesia, slow capillary refill, 3 = cool, paralysis, numb/insensate). Location of ischaemia is defined by the proximal site of symptoms on initial neurovascular assessment

*One patient experienced degloving to both the leg and foot

**One patient required fasciotomies to both the leg and thigh

Damage to the skin, nerves and muscle also occurred. Five patients had degloving injuries (avulsion of the skin with an intact musculoskeletal unit). Two patients suffered limb ischaemia equivalent to a score of 2 points on the Mangled Extremity Severity Score (MESS) and 6 patients suffered limb ischaemia equivalent to a score of 3 points. There were seven cases of rhabdomyolysis and four cases of compartment syndrome. The peak creatine kinase was 1620 U/L. Rhabdomyolysis and compartment syndrome were more common amongst individuals with fractures of the thigh and leg compared to the foot. Two patients suffered with pneumothorax and one with haemothorax.

### Management of crush injuries

The medical and surgical management is shown in Table 3. 17 patients required fluid resuscitation with intravenous sodium chloride or compound sodium lactate (Hartmann’s solution). 15 patients received Hartmann’s and six patients were resuscitated with sodium chloride. 19 patients received intravenous antibiotics. Five patients required a blood transfusion. One patient each received intravenous sodium bicarbonate and intravenous mannitol infusion.

**Table 3:** Management of crush injuries

Management		Number of patients (n = 27)
*Intravenous management*		
Fluid resuscitation	Total	17
	Hartmann’s	11
	0.9% Sodium chloride	2
	Hartmann’s and 0.9% Sodium chloride	4
Antibiotics		19
Blood transfusion		5
Urinary alkalinisation with sodium bicarbonate		1
Mannitol infusion		1
*Surgical management*		
Individuals requiring surgery		18
Above knee amputation		1
Through knee amputation		2
Below knee amputation		6
Fasciotomy		4
Closure of fasciotomy		2
Skin graft		5
Skin flap		1
Tendon repair		1
ORIF Tibia		3
Suprapatellar nailing of tibia		2
ORIF Femur		1
ORIF ankle		1
Sacroiliac joint fixation		1
K-wire fixation of toe/foot		3
Wound exploration/Washout/Debridement/Vacuum-assisted closure		11
Total number of operations		58

*ORIF* Open reduction and internal fixation

In total, 58 operations were performed on the 18 patients. Decisions on surgical management were undertaken after multidisciplinary input from orthopaedic, vascular and plastic surgeons. Ten amputations were performed in total (two above knee, two through knee and six below knee) on seven patients. One individual had bilateral amputations and two individuals required revision knee amputations. Four patients required fasciotomies. The timing of operations is shown in Table 4. All fasciotomies were performed within 12 h and two fasciotomy wounds were closed within 48 h. Four amputations were performed within 12 h, whereas five amputations were performed after 48 h. 11 procedures were carried out for fracture fixation, including three K-wire fixations of the foot, five open reduction and internal fixations (ORIF), two suprapatellar nails, and one sacroiliac joint screw insertion. Six patients required skin grafts or flaps. Skin grafts were all performed in the delayed setting (> 48 h after admission).

**Table 4:** Timing and site of operations

Operation	Site	Number of operations		
		Immediate (< 12 h)	Early (< 48 h)	Delayed (> 48 h)
Fasciotomy	Thigh	1	0	0
	Leg	3	0	0
	Foot	1	0	0
Amputation	Above knee	1	0	1 (3 days)
	Through knee	1	0	1 (5 days)
	Below knee	2	1	3 (median 4 days)
Skin graft	Amputation stump	0	0	3 (median 7 days)
	Foot	0	0	2 (7 days, 93 days)
Skin flap	Leg	0	0	1 (6 days)
Closure of fasciotomy	Thigh	0	1	0
	Leg	0	1	0

### Complications of crush injuries

Five patients experienced complications of crush injuries (Table 5). The median age was 58, two patients had type II diabetes mellitus and one patient had hypertension and a previous myocardial infarction complicated by atrial fibrillation and congestive heart failure. Of those with complications, four patients had suffered leg injuries, including one with injuries to the ipsilateral thigh and one with bilateral thigh and leg injuries. One patient had crushing of the foot and ankle. Notably, no patients with isolated foot injuries suffered systemic complications and four out of five had experienced rhabdomyolysis. This suggests, as expected, that the cohort experiencing the complications of ‘crush syndrome’ had a more severe injury profile. Three patients had AKI (two of whom had suffered rhabdomyolysis), two patients had hyperkalaemia, one patient had metabolic acidosis, and four patients suffered from sepsis. Median hospital stay among those suffering complications was 45 days, compared to six days for the whole cohort. One patient died during their hospital admission. The cause of death was severe acute respiratory syndrome coronavirus 2 (SARS-CoV-2) pneumonitis on a background of AKI caused by rhabdomyolysis. No patients showed ECG changes consistent with hyperkalaemia, and there were no episodes of acute arrhythmia. The peak serum potassium was 6.6 mmol/L. No patients required dialysis or haemofiltration.

**Table 5:** Complications of crush injuries

Complication	Number of patients (n = 5)		
	< 48 h	< 7 days	> 7 days
Acute kidney injury	2	0	1
Hyperkalaemia	1	1	0
Metabolic acidosis	1	0	0
Sepsis	0	2	2
Acute respiratory distress syndrome	0	0	0
Death	0	0	1
Median length of hospital stay, days	45		

## Discussion

Crush injuries, although commonly reported in the disaster setting [6, 7, 14], are less common in civilian settings. We identified 27 patients with crush injuries to the lower limbs and described the mechanisms, injury sites, musculoskeletal injuries, and management.

Severe crush injuries of the lower limbs caused by vehicles and machinery have been described in the industrial [17–19] and roadside [20–22] settings. Myerson et al. found crush injuries of the foot to be associated with prolonged morbidity and treatment according to a standardised protocol could reduce injury severity [15]. Nicholls et al. found the most common mechanism of crush injury to the foot in the non-disaster settings to be weight drop, followed by runover by a vehicle over a ten-year period [16]. More than two-thirds of the crush injuries in our cohort involved vehicles and seven patients were crushed by falling objects, often in the industrial setting. Michaelson described a crush injury as a continuous prolonged pressure on the limbs in patients extricated after being trapped for at least four hours [23]. Crush injury in the non-disaster setting more closely parallels the definition given by Greaves et al. where the severity of the condition is related to the magnitude and duration of the compressing force, and the bulk of muscle affected, but is not dependent on the duration of force applied [3]. In road traffic accidents we expect a large force experienced for a short duration, as in the case of the ten patients who were run over in our study. Fewer patients are likely to experience prolonged extrication in the civilian setting; in our study one patient was trapped inside a vehicle and two patients were trapped under furniture.

The likelihood of developing AKI is said to be proportional to the magnitude of the crushing force, the duration of crush, and the mass of skeletal muscle crushed [3, 23, 24]. Muscle can survive ischaemia for up to four hours, but violent crushing causes immediate muscle death [25]. Although, Oda et al. reported no correlation between time trapped and severity of crush syndrome [26], Demirkiran et al. reported higher rates of mortality and renal failure in those who were trapped for more than 24 h [6]. However, patients crushed briefly by a large force, as in this study, do not often go on to develop crush syndrome [5]. We were unable to determine the duration of crush injury in our patients, which represents a limitation of observational data collection.

Injury burden differed according to site of injury. Only patients with injury to the leg or thigh suffered bilateral leg injuries and fractures to the pelvis, ribs, and spine. In contrast, there were no bilateral foot injuries. Systemic complications of crush injuries were also limited to those with crushing of the leg or thigh and four of five patients with systemic complications suffered from rhabdomyolysis. This suggests, features of crush syndrome correlate with the severity of musculoskeletal injury, as expected. Three patients suffered from AKI. This is considerably lower than the incidence described in natural disasters [26]. In the setting of crush injury, AKI is due to the nephrotoxic myoglobinuric insult as well as a possible hypovolaemic prerenal cause. No patients with isolated foot injuries suffered from rhabdomyolysis; injuries to the legs increase the possibility of traumatic rhabdomyolysis due to the greater skeletal muscle mass compared to the foot. Hyperkalemia is one of the most important complications of rhabdomyolysis [5, 27] and two patients in our study suffered from hyperkalaemia. Although ARDS requiring mechanical ventilation has been described in the post-earthquake setting, no patient suffered from ARDS in our study [7]. Crush victims are susceptible to developing severe sepsis due to infection from trauma, surgical sites, and intravenous cannulas, and sepsis is associated with very poor survival rates [5]. 19 patients received intravenous antibiotics and four individuals, all with crush injuries to the leg or thigh, suffered from sepsis. Of the four patients with sepsis, two suffered open fractures, three suffered rhabdomyolysis and two required primary amputation, suggesting sepsis was a feature of severely injured patients.

Aggressive fluid resuscitation is the mainstay of management of crush injuries and aims to prevent the development of AKI [9]. Greaves et al. have suggested an initial bolus of 2 L of crystalloid should be given, followed by 1–1.5 L per hour, with a preference for saline [3]. Administration of warmed crystalloid helps to reverse metabolic acidosis and coagulopathy and prevents prerenal, renal and postrenal kidney injury [5]. 17 patients required fluid resuscitation. Despite the theoretical disadvantage of exacerbating hyperkalaemia, Hartmann’s solution was given in the majority (n = 11) of cases where intravenous fluids were required. Alkalinisation of the urine using sodium bicarbonate can prevent pigment nephropathy and the need for dialysis [5]. Greaves et al. have suggested maintaining urine pH above 6.5 with sodium bicarbonate [3]. Chunguang et al. described all patients receiving alkalinisation [7], whereas only one patient received sodium bicarbonate in our study. Brown et al. suggested that alkaline diuresis is only beneficial in preventing dialysis or mortality if initial serum CK levels are greater than 30,000 U/L and that a serum CK > 5000 U/L was the best prognostic marker for development of acute kidney injury [28]. In our study the peak serum CK was 1620 U/L, which is minimal in comparison to the 278,000 U/L described in crush injuries after the Sichuan earthquake [7]. Thus, we suggest that crush injuries in the civilian setting are less likely to reach a clinically significant threshold to warrant alkaline diuresis compared to natural disasters. This may be due to the faster access to pre-hospital and hospital care as well as the nature and extent of injuries in this population.

Leg amputations are common in the setting of crush injury. Chunguang et al. reported all of their patients underwent limb amputations after the Sichuan earthquake [7]. Seven of our patients underwent amputation, one patient required bilateral amputation and two required revision amputations. There were seven primary amputations performed and one amputation was performed after failed limb salvage. Amputations were required where limbs were deemed to be unsalvageable by clinical judgement; five limbs had irreparable damage to soft tissue and bone, two had compromised blood supply, and one had injury to soft tissue, bone, and blood vessels. The remaining two amputations were performed due to residual necrotic tissue.

Compartment syndrome occurs due to uptake of large amounts of fluid into damaged muscle tissue within the restricted compartment, resulting in tissue ischaemia. Boukloch et al. found that crush injuries are associated with an increased likelihood of acute compartment syndrome [29]. Debate exists around performing fasciotomy, with prevention of muscle necrosis [30] balanced against the possibility of wound infection [31]. Prophylactic fasciotomy is not recommended in crush syndrome. Greaves et al. have suggested that decompression with mannitol should be the initial treatment, followed by fasciotomy in refractory cases [3, 32]. Fasciotomy was performed in four patients, where the clinical evidence of compartment syndrome outweighed the risk of potential infection. Fasciotomies of the leg were performed according to BOAST guidelines (two-incision four-compartment decompression) [33]. Foot fasciotomy was performed with two dorsal incisions, one medial and one lateral incision. Medial and lateral incisions were used in the thigh. Management of fasciotomy wounds is an important consideration in the crush injury patient. Two patients underwent primary closure of fasciotomy wounds within 48 h, with one requiring subsequent amputation. One patient underwent amputation without fasciotomy closure and one patient required a skin graft for closure. Fasciotomy may result in long term dysaesthesia, weakness and soft tissue contractures. Rehabilitation after fasciotomy therefore includes wound protection, early mobilisation and weight bearing, physiotherapy support, passive and active stretching of joints and sensory re-education [34].

There are some limitations to this study. The retrospective design means incorrectly coded injuries may have been missed. Furthermore, as crush injuries are rare, we can only draw our conclusions from a small patient cohort which is not suitable for statistical comparisons. The rarity of crush injuries combined with the range of severity at presentation resulted in heterogeneity of the initial clinical assessment. A standardised crush injury protocol with the use of clinical severity measures (e.g. MESS) would further elucidate the relationship between injury severity and risk of developing crush syndrome in the non-disaster setting. Nevertheless, our detailed descriptions of the cohort of injuries highlight the key injury and treatment factors for this group of patients.

## Conclusion

Crush injuries are rare. In our study, we identified 27 individuals with crush injuries to the lower limbs over an eight year period. Mechanisms of injury were distinct from those seen in disaster settings, with road-traffic and industrial accidents being common. Severity of injury was not uniform; 16 patients suffered fractures, whereas four suffered compartment syndrome. A majority of patients received intravenous fluid resuscitation and antibiotics and seven patients required amputations. Although seven patients suffered rhabdomyolysis, severity of crush injury in the non-disaster setting may be insufficient to warrant routine alkaline diuresis. A reduced burden of musculoskeletal and soft tissue injury, need for fluid resuscitation, and surgical burden was observed in those with isolated foot injuries. Furthermore, only patients with injuries to the leg or thigh experienced complications resembling crush syndrome. Thus, these patients more closely resemble the patient cohort seen in natural disasters compared to individuals with an isolated crush injury to the foot.

## Data Availability

Anonymised data available on request.
